# Graves’ disease coexisted with resistance to thyroid hormone: a case report

**DOI:** 10.1186/s13256-021-03061-4

**Published:** 2021-09-25

**Authors:** Hiroshi Akahori, Rika Usuda

**Affiliations:** 1 Department of Endocrinology and Metabolism, Toyama Prefectural Central Hospital, 2-2-78 Nishinagae, Toyama-shi, Toyama, 930-8550 Japan; 2 Department of Endocrinology, Diabetology and Metabolism, Toyama Prefectural Rehabilitaion Hospital and Support Center for Children with Disabilities, 36 Shimo-Iino, Toyama-shi, Toyama, 931-8517 Japan

**Keywords:** Graves’ disease, Resistance to thyroid hormone, Thyroid hormone receptor β, R282S, Syndrome of inappropriate secretion of TSH

## Abstract

**Background:**

Resistance to thyroid hormone is a rare autosomal dominant disorder characterized by reduced responsiveness to thyroid hormone and can cause syndrome of inappropriate secretion of thyroid stimulating hormone. Although Graves’ disease is a common autoimmune thyroid disorder, the coexistence of these two diseases is extremely rare and makes the diagnosis and treatment complicated, leading to the delayed diagnosis of resistance to thyroid hormone. We describe the case of a Japanese man with resistance to thyroid hormone coexisting with Graves’ disease, in which the correct diagnosis of resistance to thyroid hormone was delayed by masking of the signs of syndrome of inappropriate secretion of thyroid stimulating hormone, with final diagnosis 30 years after the initial treatment for Graves’ disease.

**Case presentation:**

A 30-year-old Japanese man presented with diffuse goiter and thyrotoxicosis. Anti-thyroid stimulating hormone receptor antibody was positive. He was diagnosed with Graves’ disease. Anti-thyroid medication was chosen as the initial treatment for Graves’ disease. However, this treatment failed to normalize the free triiodothyronine, free thyroxine, and thyroid stimulating hormone levels. His thyroid hormone levels indicated syndrome of inappropriate secretion of thyroid stimulating hormone. After cessation of methimazole treatment by remission of Graves’ disease, his state of syndrome of inappropriate secretion of thyroid stimulating hormone persisted. Magnetic resonance imaging revealed no pituitary tumor lesions. The results of thyroid stimulating hormone-releasing hormone stimulation test showed a normal response of thyroid stimulating hormone. He was suspected to have resistance to thyroid hormone. Direct sequencing analysis of the thyroid hormone receptor β gene identified a heterozygous missense mutation, R282S. Coexistence of resistance to thyroid hormone with Graves’ disease was confirmed. He has no signs of thyrotoxic symptoms, and is capable in activities of daily living at the present time.

**Conclusion:**

We described a rare case of resistance to thyroid hormone simultaneously existing with Graves’ disease. This case demonstrated that these diseases can coexist, and indicated some of the difficulties in diagnosis of resistance to thyroid hormone with coexisting Graves’ disease. The diagnosis of resistance to thyroid hormone did not become apparent until after anti-hyperthyroidism treatment. Although rare, careful follow-up after the initial treatment of Graves’ disease is necessary. The coexistence of these two diseases should be considered in patients showing occasional syndrome of inappropriate secretion of thyroid stimulating hormone.

## Background

Resistance to thyroid hormone (RTH) is an autosomal dominant disorder characterized by reduced responsiveness of variable tissues to thyroid hormone [1, 2]. The human thyroid hormone receptor (TR) is expressed by two genes, TRβ and TRα, which have several isoforms generated by alternative splicing. TRβ1 is expressed in the liver, kidney, and brain, while TRβ2 is expressed in the anterior pituitary and hypothalamus, and is mainly involved in the negative feedback regulation of thyroid stimulating hormone (TSH) by thyroid hormone in the anterior pituitary. RTH is mainly caused by germline mutations of TRβ, localized to the ligand-binding domain [3–5]. The mutant receptors show reduced ligand binding, and inhibit wild-type TR action in a dominant negative manner [6]. This syndrome was first reported by Refetoff *et al*. in 1967 [7]. The syndrome of inappropriate secretion of TSH (SITSH) showing elevated serum levels of free thyroxine (FT4) and free triiodothyronine (FT3) with normal or slightly elevated TSH levels (non-suppressed TSH) is a hallmark of RTH as well as TSH producing pituitary tumors [8].

Graves’ disease is a common autoimmune thyroid disorder, which is responsible for approximately 50–80% of cases of thyrotoxicosis in Japan. In Graves’ disease, the circulating immunoglobulin G antibodies bind to the G-protein-coupled TSH receptor, and increase thyroid hormone production, causing hyperthyroidism. This disorder shows elevated FT3 and FT4 with suppression of TSH to below the level of detection, as opposed to RTH.

Thus, the coexistence of these two diseases makes the diagnosis and treatment complicated. Here, we describe a rare case of RTH coexisting with Graves’ disease, in which the correct diagnosis of RTH was delayed by masking of the signs of SITSH, with final diagnosis due to the inappropriately elevated TSH level after treatment of anti-hyperthyroidism.

## Case presentation

**Fig. 1:**
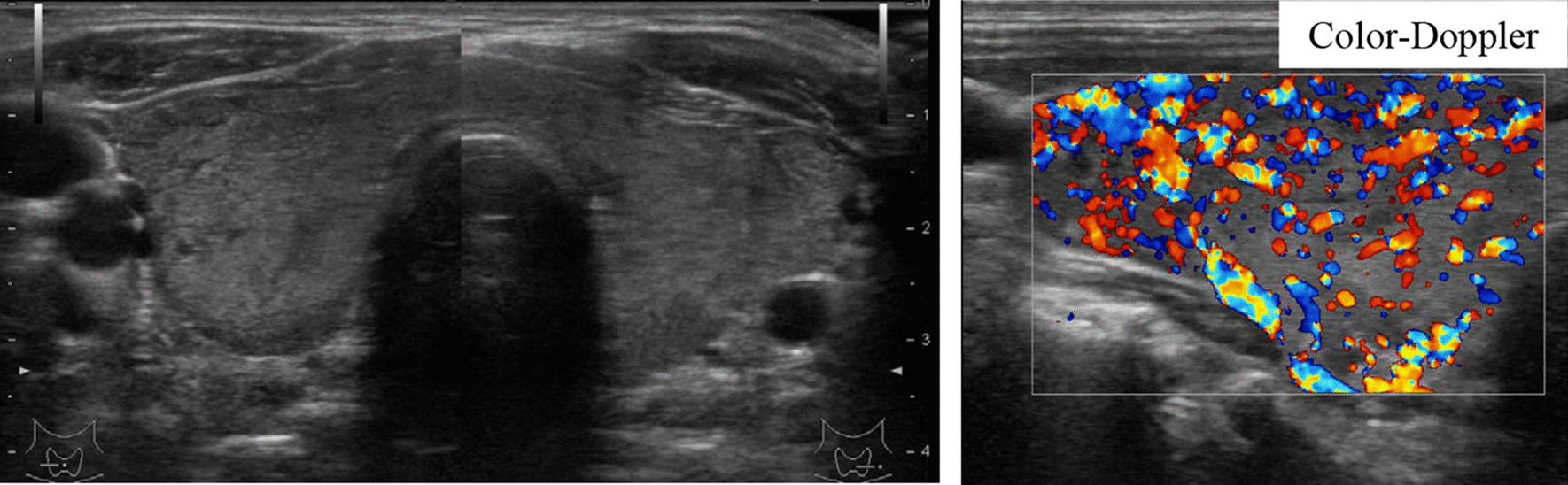
Thyroid ultrasonography showed diffuse enlargement of both lobes with heterogeneous echogenicity. No tumor lesions were detected. Neither lobe showed increased blood flow on Color-Doppler imaging

**Fig. 2:**
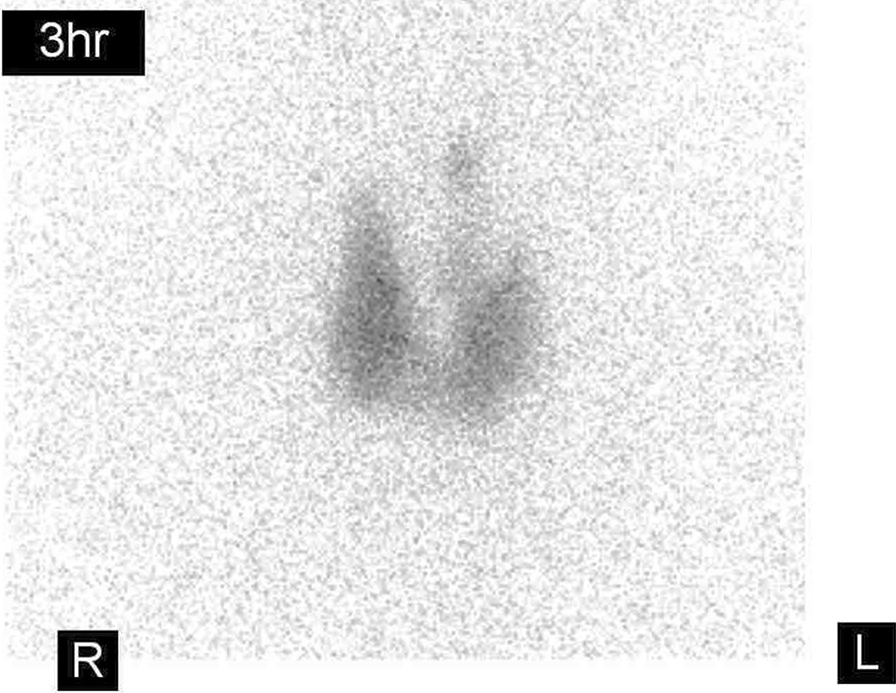
Thyroid radioactive ^123^I uptake showed no increase. The uptake was 11.8% in 3 hours (reference range, 5–15%)

**Fig. 3:**
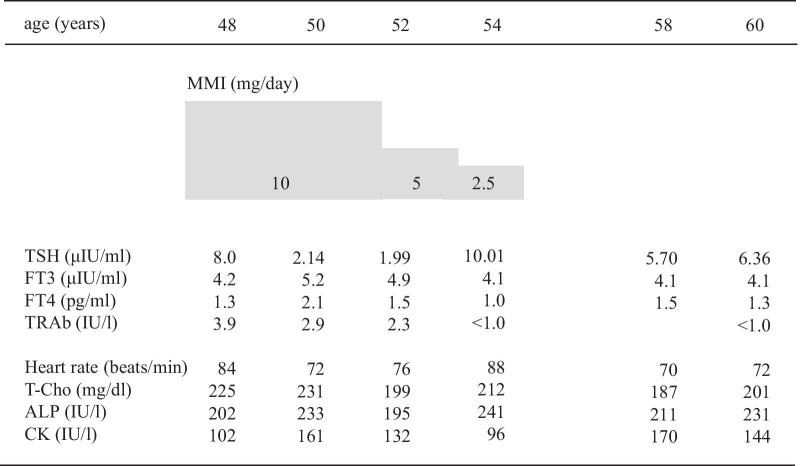
Clinical course after anti-hyperthyroidism treatment for Graves’ disease. Thyroid hormone levels showed occasional SITSH in the periods of anti-hyperthyroidism treatment. Although this treatment led to remission of Graves’ disease, it failed to normalize the FT3, FT4, and TSH levels. SITSH persisted even after cessation of methimazole treatment

**Fig. 4:**
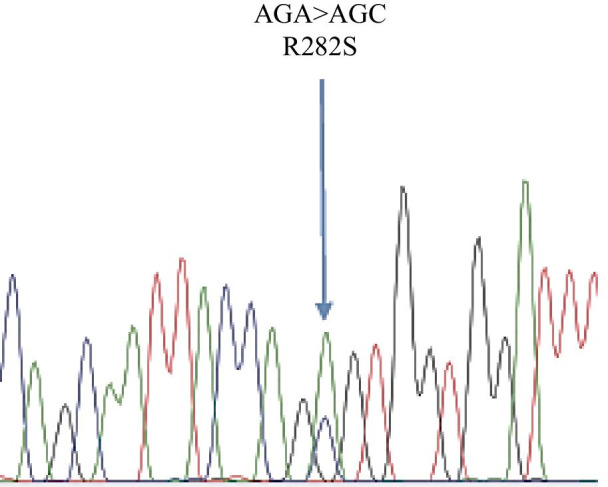
Direct sequencing analysis of TRβ gene in genomic DNA extracted from peripheral blood leukocytes. Sequence analysis revealed a heterozygous point mutation in exon 8, AGA to AGC, resulting in substitution of arginine by serine at codon 282 (R282S). The arrow indicates the site of the point mutation

A 30-year-old Japanese man was referred to our hospital because of fatigue, palpitation, body weight loss, finger tremor, and heat intolerance for 3 months. He was 166.2 cm in height and 60.2 kg in weight. His blood pressure was 110/50 mmHg, and his heart rate was 70 beats/minute and regular. His body temperature was 36.3 °C. The thyroid gland was slightly enlarged, non-tender, and softly elastic on palpation. Exophthalmos was not observed. Thyroid function test demonstrated elevated levels of FT3 and FT4, and complete suppression of TSH level, which was below the limit of detection. Serum anti-TSH receptor antibody (TRAb), anti-thyroid peroxidase antibody (TPOAb), and anti-thyroglobulin antibody (TgAb) were positive. Ultrasonography of the thyroid gland revealed diffuse enlargement without tumor lesions. The right and left lobes of the thyroid gland measured 49 × 24 × 20 mm and 54 × 22 × 20 mm, respectively. These results indicated that Graves’ disease was responsible for primary hyperthyroidism in this case. A diagnosis of Graves’ disease was made based on these findings. Methimazole (MMI) therapy was started at 15 mg/day. His symptoms improved gradually, and the dosage of methimazole was decreased as thyroid function normalized. After 6 months, methimazole therapy was continued at 10 mg/day, and the levels of TSH, FT3, and FT4 were maintained within the respective normal ranges. He was transferred to a local hospital. His thyroid function was followed up periodically, and the levels of FT3 and FT4 were elevated, but TSH levels were within normal range or slightly elevated on some occasions. At the age of 48 years, he was referred to our hospital for further endocrinological examination. On physical examination, his height was 165.4 cm, body weight was 58.6 kg, body mass index was 22.1 kg/m^2^, blood pressure was 122/70 mmHg, and his pulse was regular at 80 beats/minute. His body temperature was 36.8 °C. The thyroid was diffusely enlarged, softly elastic, and non-tender on palpation. Exophthalmos was not observed. Laboratory findings are presented in Table 1. The results of thyroid function tests with methimazole treatment at 10 mg/day revealed serum levels of FT3, FT4, and TSH of 5.4 pg/mL (normal range 2.4–4.3 pg/mL), 1.7 ng/dL (normal range 0.9–1.8 ng/dL), and 4.77 μIU/mL (normal range 0.35–4.94 μIU/mL), respectively, indicating SITSH. Chest X-ray and electrocardiographic findings were normal. His bone mineral density was 0.712 g/cm^2^ (88% of the young adult mean) in the femur. He showed no stigmata of thyrotoxicosis. Ultrasonography of the thyroid showed diffuse enlargement of both lobes with no tumor lesions. Increased blood flow was not detected on Color-Doppler imaging (Fig. 1). Thyroid radioactive ^123^I uptake was 11.8% in 3 hours (reference range, 5–15%) (Fig. 2). The titer of TRAb was weakly positive, and the dosage of methimazole was decreased gradually to maintain normal TSH level (Fig. 3). Six years after he visited our hospital, methimazole treatment was ceased, and the titer of TRAb became negative. These findings indicated that Graves’ disease was in remission. The symptoms of hyperthyroidism, such as finger tremors, palpitations, and weight loss, were not observed. After cessation of methimazole treatment, his thyroid hormone levels fluctuated and showed occasional elevation of FT3 or FT4 with normal TSH level. His state of SITSH persisted. Magnetic resonance imaging (MRI) was performed for the differential diagnosis of TSH producing pituitary tumor, and revealed no tumor lesions. The results of TSH-releasing hormone (TRH) stimulation test are presented in Table 2. His TSH level showed a normal response to TRH stimulation. He was suspected to have RTH. A genetic investigation was conducted with the patient’s written informed consent. Blood samples were obtained, and genomic DNA was isolated from leukocytes. Direct sequencing analysis of the TRβ gene identified a heterozygous, missense point mutation in exon 8, AGA to AGC, resulting in substitution of arginine by serine at codon 282 (R282S) (Fig. 4). He reported no thyroid disease in his family. Thyroid function tests and DNA analysis were not performed for other family members, because they declined to provide consent.

**Table 1:** Laborator﻿y data on arrival

*Urinalysis*		*Biochemistry*		*Endocrinological examination*		
Color	Yellow	LDH	181 IU/L	TSH	4.77 μIU/mL	(0.35–4.94)
pH	6.5	ALP	191 IU/L	FT3	5.4 pg/mL	(2.4–4.3)
Glucose	(−)	γ-GTP	33 IU/L	FT4	1.7 ng/dL	(0.9–1.8)
Protein	(−)	CK	151 IU/L	Tg	3.0 ng/mL	(0–33.7)
Occult blood	(−)	LDL-C	121 mg/dL	TRAb	3.9 IU/L	(0–1.0)
Ketone	(−)	TG	146 mg/dL	TSAb	269%	(0–120)
		HDL-C	48 mg/dL	TPO Ab	536 IU/mL	(0–16.0)
*Hematology*		Na	140 mEq/L	Tg Ab	398 IU/mL	(0–28)
WBC	5600/μL	K	4.2 mEq/L	ACTH	18.4 pg/mL	(7.2–63.3)
RBC	485 × 10^4^/μL	Cl	102 mEq/L	cortisol	9.9 μg/dL	(4.5–21.1)
Hb	13.5 g/dL	Ca	9.4 mEq/L	GH	0.48 ng/mL	(0.13–9.88)
Ht	45.60%	P	4.9 mEq/L	IGF-1	127 ng/mL	(89–248)
Plts	26.4 × 10^4^/μL	BUN	16 mg/dL	PRL	7.72 ng/mL	(3.6–12.8)
CRP	0.1 mg/dL	Cr	0.7 mg/dL	FSH	6.74 mIU/mL	(2.0–8.3)
TP	7.5 g/dL	UA	6.7 mg/dL	LH	5.29 mIU/mL	(0.8–5.7)
Alb	4.5 g/dL	FPG	81 mg/dL			
T-Bil	0.47 mg/dL	HbA1c	5.50%			
AST	22 IU/L					
ALT	19 IU/L					(Normal range)

γ-GTP: γ-glutamyl transpeptidase; ACTH: adrenocorticotropic hormone; Alb: albumin; ALP: alkaline phosphatase; ALT: alanine aminotransferase; AST: aspartate aminotransferase; BUN: blood urea nitrogen; CK: creatine kinase; Cr: creatine; CRP: C-reactive protein; FPG: fasting plasma glucose; FSH: follicle stimulating hormone; FT3: free triiodothyronine; FT4: free thyroxine; GH: growth hormone; Hb: hemoglobin; HbA1c: glycated hemoglobin; HDL-C: high-density lipoprotein-cholesterol; Ht: hematocrit; IGF-1: insulin-like growth factor-1; LDH: lactate dehydrogenase; LDL-C: low-density lipoprotein-cholesterol; LH: luteinizing hormone; Plts: platelets; PRL: prolactin; RBC: red blood cells; T-Bil: total bilirubin; TG: triglyceride; Tg: thyroglobulin; Tg Ab: anti-thyroglobulin antibody; TP: total protein; TPO Ab: anti-thyroid peroxidase antibody; TRAb: anti-TSH receptor antibody; TSAb: thyroid-stimulating antibody; TSH: thyroid-stimulating hormone; UA: uric acid; WBC: white blood cells

**Table 2:** Thyrotropin-releasing hormone stimulation test

Time (minutes)	Basal	30	60	90	120
TSH (μIU/mL)	7.24	37.5	33.3	24.0	17.4
FT3 (pg/mL)	4.19				4.40
PRL (ng/mL)	7.16	88.03	51.12	29.90	20.89

FT3: free triiodothyronine; PRL: prolactin; TSH: thyroid-stimulating hormone

At the time of writing, he has no signs of thyrotoxic symptoms. His pulse rate is regular at 80–90 beats/minute. He is comfortable at rest, and capable in activities of daily living.

## Discussion and conclusions

RTH is a rare autosomal dominant disorder characterized by reduced responsiveness to thyroid hormone, and is estimated to affect approximately 1 per 40,000 newborn infants [9]. Thyroid hormones have roles in a range of functions, including energy homeostasis, cardiac and gastrointestinal function, and maturation of the central nervous system. These actions are mediated by TRs encoded by two genes: TRβ and TRα. This syndrome is associated with germline mutations of TRβ. TRβ gene encodes two isoforms by alternative splicing, TRβ1 and TRβ2. TRβ1 is widely expressed, especially in the liver and kidney. TRβ2 is expressed in the anterior pituitary and hypothalamus, and mainly contributes to the negative feedback regulation of TSH by thyroid hormone in the anterior pituitary. RTH is associated with genetic mutations in the ligand-binding domain of TRβ, which is common between TRβ1 and TRβ2. Thus, mutations of TRβ cause failure of this negative feedback regulation of TSH by thyroid hormone, and can cause SITSH. The pathogenesis of RTH is related to the distributions of TRβ and TRα in different tissues, and dominant negative activity of mutant receptors on different target genes. Resistance to thyroid hormone is compensated by increased thyroid hormone levels. Therefore, the patients with RTH have no severe symptoms of hypothyroidism.

Here, we reported a Japanese man with RTH concomitant with Graves’ disease. In the onset of Graves’ disease, his TSH values were suppressed below the limit of detection. Furthermore, he showed the apparent features of hyperthyroidism, such as general fatigue, palpitation, body weight loss, finger tremor, and heat intolerance. These observations indicated that peripheral tissues were responsive to excess thyroid hormone. Therefore, the differential diagnosis between Graves’ disease alone and Graves’ disease coexisting with RTH is very difficult after the onset of hyperthyroidism by Graves’ disease. Our case was diagnosed with RTH from the presentation of SITSH after anti-hyperthyroidism treatment. The presentation of SITSH indicates the possibility of RTH or TSH-producing pituitary adenoma [10]. In our case, brain MRI showed no pituitary adenoma, and TRH stimulation test showed a normal response to TSH, thus excluding TSH-producing pituitary adenoma. We analyzed the TRβ mutations in our case, and the results showed that he carried a heterozygous missense mutation, R282S.

Treatment for Graves’ disease with RTH is complicated. Three cases with both Graves’ disease and RTH have been reported previously [11–13]. In these cases, as well as our case, anti-thyroid medication was chosen as the initial treatment for Graves’ disease. In our case, anti-thyroid medication led to remission of Graves’ disease. However, this treatment failed to normalize the FT3, FT4, and TSH levels, due to the failure of the negative feedback regulatory system by RTH. Patients with RTH usually have elevated levels of FT3 and FT4 and normal or slightly elevated TSH level, although the thyroid function before the onset of Graves’ disease was unknown in our case. If the levels of FT3 and FT4 are within the normal reference ranges, the patient may have a hypothyroid state. Therefore, the dosage of anti-thyroid medication should be controlled to maintain slightly elevated levels of FT3 and FT4. The relationship between RTH and Graves’ disease is unclear. Cases of RTH coexisting with chronic thyroiditis, not only Graves’ disease, have been reported [14–16]. It has also been reported that patients with RTH have high prevalence of TPOAb and TgAb, indicating the potential interactions between RTH and autoimmune thyroid disease [17]. Moreover, the hypersecretion of TSH may play an important role in the onset of Graves’ disease. TSH has various important actions in the maintenance of normal physiology and in the regulation of immunomodulatory gene expression of thyrocytes. Patients with RTH show elevated production of TSH. It has been reported that the abnormal hypersecretion of TSH in thyrotropin-producing pituitary adenoma can produce anti-idiotype antibodies, causing Graves’ disease [18–20]. In our case, the elevated TSH levels due to failure of this negative feedback regulation of TSH by thyroid hormone may have produced TRAb and induced the onset of Graves’ disease. The further accumulation of clinical data will be necessary to determine whether germline mutations of TRβ induce autoimmune thyroid disease in patients with RTH.

In conclusion, we described a rare case of RTH simultaneously existing with Graves’ disease. The patient carried a mutation, R282S, in the TRβ gene. This case indicated some of the difficulties in diagnosis of RTH with coexisting Graves’ disease. The diagnosis of RTH did not become apparent until after anti-hyperthyroidism treatment. Careful follow-up is necessary to determine whether secretion of TSH may be inappropriate to regulate thyroid hormone in the periods of anti-hyperthyroidism treatment. The coexistence of these two diseases should be considered in patients showing occasional SITSH.

## Data Availability

All data and materials are available from the corresponding author on reasonable request.

## Abbreviations

γ-GTP: γ-Glutamyl transpeptidase
ACTH: Adrenocorticotropic hormone
Alb: Albumin
ALP: Alkaline phosphatase
ALT: Alanine aminotransferase
AST: Aspartate aminotransferase
BUN: Blood urea nitrogen
CK: Creatine kinase
CPR: C-peptide immunoreactivity
Cr: Creatine
CRP: C-reactive protein
FPG: Fasting plasma glucose
FSH: Follicle stimulating hormone
FT3: Free triiodothyronine
FT4: Free thyroxine
GH: Growth hormone
Hb: Hemoglobin
HbA1c: Glycated hemoglobin
HDL-C: High-density lipoprotein cholesterol
Ht: Hematocrit
IGF-1: Insulin-like growth factor-1
LDH: Lactate dehydrogenase
LDL-C: Low-density lipoprotein cholesterol
LH: Luteinizing hormone
MMI: Methimazole
MRI: Magnetic resonance imaging
Plts: Platelets
PRL: Prolactin
RBC: Red blood cells
RTH: Resistance to thyroid hormone
SITSH: Syndrome of inappropriate secretion of TSH
T-Bil: Total bilirubin
TG: Triglyceride
Tg Ab: Anti-thyroglobulin antibody
TP: Total protein
TPO Ab: Anti-thyroid peroxidase antibody
TR: Thyroid hormone receptor
TRAb: Anti-TSH receptor antibody
TSAb: Thyroid-stimulating antibody
TSH: Thyroid-stimulating hormone
UA: Uric acid
WBC: White blood cells
